# Acupuncture for Chronic Prostatitis or Chronic Pelvic Pain Syndrome: An Updated Systematic Review and Meta-Analysis

**DOI:** 10.1155/2023/7754876

**Published:** 2023-03-14

**Authors:** Juanhong Pan, Song Jin, Quan Xie, Ying Wang, Zhipeng Wu, Jianfeng Sun, Tai Pin Guo, Di Zhang

**Affiliations:** ^1^School of Health Preservation and Rehabilitation, Chengdu University of Traditional Chinese Medicine, Chengdu, Sichuan, China; ^2^Rehabilitation Department, Hospital of Chengdu University of Traditional Chinese Medicine, Chengdu, Sichuan, China; ^3^School of Medical and Life Sciences, Chengdu University of Traditional Chinese Medicine, Chengdu, Sichuan, China; ^4^Acupuncture, Tuina and Rehabilitation School of the Yunnan University of Chinese Medicine, Kunming, Yunnan, China

## Abstract

**Background:**

Chronic prostatitis/chronic pelvic pain syndrome (CP/CPPS) is a complex male dysfunction, mostly seen in young and middle-aged men with a history of more than 3 months. As a traditional therapy of Traditional Chinese Medicine, acupuncture has been proven an effective method to treat CP/CPPS in recent years. Though some meta-analyses on acupuncture for chronic prostatitis were published in 2018 and 2019, most of the included studies were low in quality according to the JADAD score (JADAD < 4). The conclusions of acupuncture for CP/CPPS remain indefinite.

**Purpose:**

This review aims to evaluate the efficacy of acupuncture for CP/CPPS by including high-quality literature only (JADAD ≥ 4) to provide a reliable basis for clinical applications and research.

**Method:**

Nine electronic databases were searched from inception to March 1, 2022, and only randomized controlled trials (RCT) with high-quality (JADAD ≥ 4) were included. Data were analyzed using Review Manager 5.3. and was verified through trial sequential analysis (TSA). We carried out a sensitivity analysis for the heterogeneity (*I*^2^ ≥ 50%). Publication bias was explored using a funnel plot.

**Result:**

Ten RCTs (11 trials) of high-quality methodology involving 798 patients were included. Meta-analysis showed that compared to sham acupuncture (SAT) and western medicine (WM), acupuncture (AT) played superior roles for CP/CPPS patients in pain score, NIH-CPSI score, quality of life score, urinary symptom, and efficacy rate. As for the adverse effects, 4 RCTs described mild hematoma and pain in AT and SAT groups, while specific symptoms including nausea, abdominal pain, dizziness, and low blood pressure were reported in WM groups.

**Conclusion:**

This meta-analysis indicated that acupuncture has measurable benefits on CP/CPPS, and security has also been ensured. However, this meta-analysis only included 10 RCTs; thus, RCTs with a larger sample size and longer-term observation are required to verify the effectiveness of acupuncture further in the future.

## 1. Introduction

Chronic prostatitis/chronic pelvic pain syndrome (CP/CPPS) is a complex neurological dysfunction, and the incidence is on the rise year by year. According to foreign reports, the incidence of prostatitis is about 9%, and the recurrence rate is 20%–50% [1]. In China, epidemiologic studies have shown that the incidence of CP/CPPS is higher than in other countries, about 6.0% to 32.9% [2–4]. Patients with CP/CPPS usually suffer from varying degrees of pelvic pain or discomfort, and lower urinary tract symptoms and most prominent in the elderly [5]. It has ranked fourth among the 20 principal diagnostic diseases in the United States [6] and the third most commonly found urinary tract disease in men [7]. Men with chronic prostatitis have low blood flow in the common iliac vein, suggesting that pelvic congestion may be related to this condition [8]. The urinary tract is naturally colonized with a specific microbiome, and any imbalance may cause impairment and functional disorders [9]. Patients with CP/CPPS often experience urinary symptoms in their filling phase, urethral pain in the voiding phase, and recurrent urinary tract infections, which severely impact the patients' quality of life. Further, evidence supports a correlation between male infertility and prostatitis, as the prostate gland is the primary male accessory gland for male fertility. Many cases are underdiagnosed and undertreated by physicians [10].

Typical therapies such as alpha-blockers, antibiotics, and anti-inflammatory medications occur with relatively modest effects on physiological functions; acupuncture may be an important consideration in difficult-to-treat pain syndromes. As a nonpharmacological intervention, acupuncture is a comprehensive concept of physical therapy that involves all kinds of needling techniques, moxibustion, and acupoint stimulation therapies. Researchers have confirmed that acupuncture is effective for CP/CPPS; however, most of the publications were nonrandomized studies [11–16]. Besides, though three similar SRs regarding acupuncture for CP/CPPS were published in 2018 [17], 2019 [18],and 2020 [19], according to the JADAD score, most of the included studies were low in quality. Furthermore, several relative trials [20–22] were conducted after the retrieval deadline (up to February, 2019) in 2020. Therefore, it is necessary to update a new SR by setting restricted inclusion and exclusion criteria and expanding search fields to draw a definite conclusion about acupuncture for CP/CPPS.

## 2. Material and Methods

### 2.1. Search Strategy

This review was reported in conformity with the Preferred Reporting Item for Systematic Review and Meta-Analyses Statement. We searched PubMed, EMBASE, Springer, ICTRP, the Cochrane Library, China National Knowledge Infrastructure (CNKI), Technology Periodical Database (VIP), Wan Fang Data, and China Biology Medicine (CBM) from the earliest available date until September 15, 2022. The following keywords were used: acupuncture (AT), chronic prostatitis/chronic pelvic pain syndrome (CP/CPPS), and randomized controlled trial (RCT). The language was restricted to Chinese and English. Study screening was performed independently by two researchers (Juanhong Pan and Di Zhang). Detailed search strategies based on guidance from the Cochrane handbook for PubMed, the Cochrane Library, and China National Knowledge Infrastructure (CNKI) would employ similar strategies for the other databases.

### 2.2. Quality of Studies

The methodological quality was assessed using an improved JADAD scale (0–3: low quality; 4–7: high-quality), and only articles with high-quality (JADAD ≥ 4) were included. Risk of bias was also performed by two reviewers (J.H.P. and D.Z.) following the Cochrane Handbook for Systematic Reviews of Interventions, Version 5.3. The items included random sequence generation (selection bias), allocation concealment (selection bias), blinding of participants and personnel (performance bias), blinding of outcome assessment (detection bias), incomplete outcome data (attrition bias), selective reporting (reporting bias), and other bias. The quality of all the included trials was categorized as low/unclear/high risk of bias (“Yes” for a low risk of bias, “No” for a high risk of bias, and “Unclear” otherwise). We evaluated every study and made judgments about potential bias.

### 2.3. Inclusion/Exclusion Criteria

The inclusion/exclusion criteria are available in the published protocols [23]. After we reviewed relevant articles, eligibility criteria for this review based on PICOS frameworks (population, intervention, comparison, outcome, and study) were as follows: (1) participants suffered from CP/CPPS; (2) broad acupuncture including traditional acupuncture (manual acupuncture, electronic acupuncture, dry needle, and scalp acupuncture) and acupuncture combination therapies (acupuncture combined with western medicine (it is medication, for example, tamsulosin capsule, levofloxacin hydrochloride or azithromycin capsules, and acupoint injection) seemed as experimental groups; (3) non -acupuncture was applied to control groups; (4) RCTs; (5) JADAD ≥ 4; and (6) study published in English or Chinese. Differences in sex, age, country, time, and race were not taken into account. Studies were excluded if they were nonrandomized studies or used other forms of acupuncture, such as transcutaneous electrical nerve stimulation and laser acupuncture. Grey literature databases and websites were not searched. There were no restrictions on population characteristics and publication type.

### 2.4. Data Extraction

Two authors (J.H.P. and D. Z.) screened the studies and collected the data independently according to the inclusion and exclusion criteria. The information of author, publication year, country (language), demographics of participants, intervention, positive or negative conclusion, treatment duration (number), treating time, frequency (per week), time (per day), follow-up, outcomes, and JADAD score were recorded. All studies were managed with Endnote X9. Disagreements were resolved by discussion or umpired with a third reviewer (Y. W.).

### 2.5. Data Synthesis

We developed inclusion/exclusion criteria for screening articles, followed by data extraction and quality evaluation. We used Review Manager 5.3 software provided by the Cochrane Collaboration for data analyses and presented the final result. For the continuous data, the mean differences (MD) and 95% confidence intervals (CI) were used when outcomes were assessed by the same scale. *I*^2^ statistical tests were adopted to assess the heterogeneity among studies. A fixed-effect model was applied to combine the data if the *I*^2^ < 50%; otherwise, a random effect model was applied. If *I*^2^ ≥ 50%, the heterogeneity was high, and sensitivity analysis would be considered. The efficacy is dichotomous data and categorized into two levels ((1) effective and (2) inefficacious). The efficacy rate means the percentage of the total number of participants categorized in the first two levels. Publication bias was explored using a funnel plot.

### 2.6. Trial Sequential Analysis

Meta-analysis usually requires multiple tests, and random errors may sometimes lead to false significant results when data are accumulated, and the increased frequency of statistical tests in a meta-analysis increases the possibility of reporting such results [24]. However, trial sequential analysis (TSA) overcomes the shortcomings of classical meta-analysis and corrects for the increase of type 1 error [25].

TSA.0.9.5.10 beta, was used for sequential analyses. If the *Z*-curve exceeds the traditional boundary but does not cross the TSA boundary, it suggests that a false positive error may be made. If it intersects the TSA boundary, it suggests that the meta-analysis results are robust, even if the RIS is not reached. The *Z* curve did not intersect with the traditional cut-off value and the TSA cut-off value, and the positive or negative conclusion could not be drawn. The *Z*-curve intersects the null line, indicating no significance [26]. We set a two-sided 5% type I error risk (*α*) and 20% type II error risk (*β*) to calculate the amount of information needed, with a 20% relative risk (RRR) reduction and a control event rate derived from data from the meta-analysis.

## 3. Results

### 3.1. Study Selection

**Figure 1:**
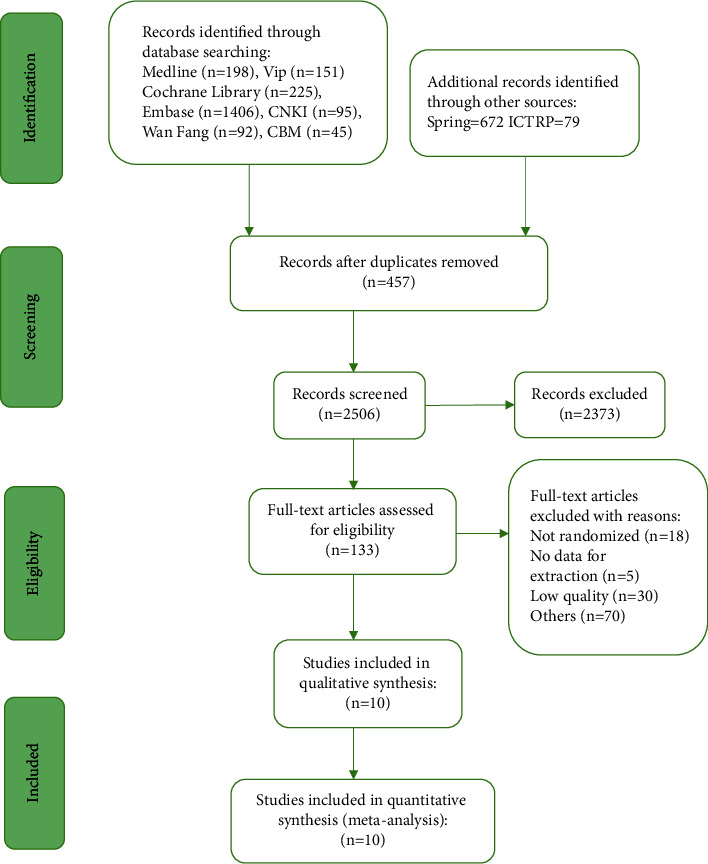
Flowchart of the selection process.

A flow chart depicts the search process and study selection (Figure 1). After primary searches from the databases, 2963 articles were screened. After reading the titles and abstracts, 2830 articles were excluded. Full texts of 133 articles were retrieved, and 123 articles were excluded with reasons listed as the following: not randomized (*n* = 18), no data for extraction (*n* = 5), low quality (*n* = 30), and others (*n* = 70). In the end, 10 RCTs were included. 5 were written by Chinese in Chinese [20, 21, 27–29], 3 [22, 30, 31] of which were written by Chinese in English, 1 by Malaysian in English [32], and 1 by Turkish in English [33].

### 3.2. Study Characteristics

The characteristics of included RCTs are listed in Tables 1 and 2. A total of 798 patients with CP/CPPS were included. There were 11 trials that specified 9 diagnostic criteria of CP/CPPS. The age, frequency, and treatment duration varied from 18 to 55 years, 1 d/w to 3 d/w, and 20 to 24 times a course, respectively. The intervention of treatment groups included scalp acupuncture, acupoint injection, electric acupuncture, acupuncture, and Tiaoshen acupuncture. The intervention of control groups included western medicine and sham acupuncture. The outcomes were NIH-CPSI, pain, urinary symptoms, quality of life, and efficacy rate.

### 3.3. Methodological Quality of Included Studies

The methodological quality of most included RCTs was generally “high” (JADAD ≥ 4), according to the quality assessment criteria with improved JADAD scale (Table 1) [20–22, 27–33]. Almost all the trials mentioned the randomized allocation of participants. Selective reporting was generally uncertain in the trials due to the inaccessibility of the trial protocol.

### 3.4. Risk of Bias

**Figure 2:**
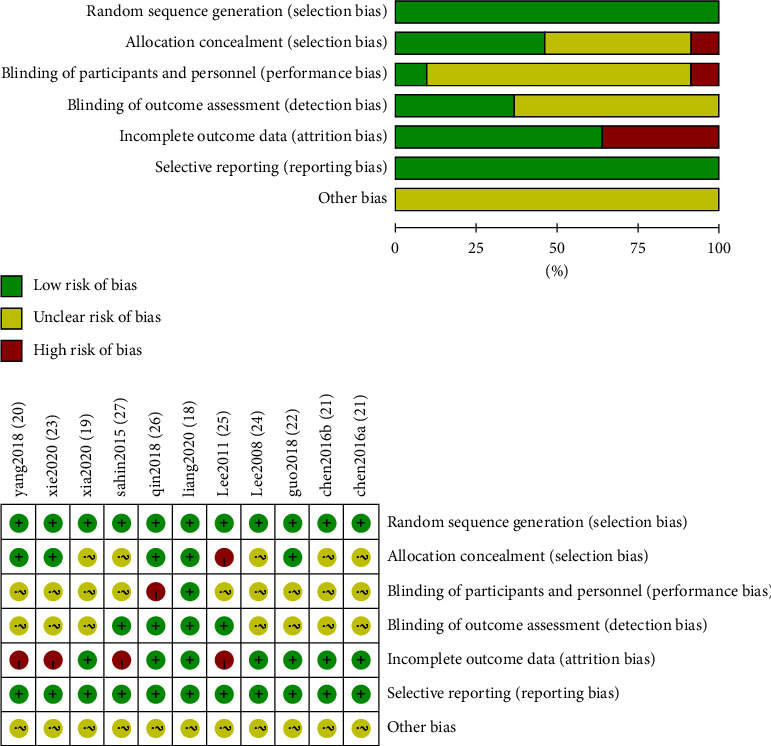
Risk of bias of included studies.

As shown in Figure 2, the 11 trials were at low risk. All of 11 trials reported random sequence generation and were assessed as low risk. 5 trials [21, 28, 32, 33] were assessed as unclear risk, 5 were assessed as low risk [20, 22, 28, 29, 31], and 1 [30] was assessed as high risk in the aspect of allocation concealment. In blinding of participants and personnel, 9 trials [21, 27–33]were assessed as unclear risk, 1 trial [20] was assessed as low risk, and 1 trial [31] was assessed as high risk. Also, 7 [21, 22, 27–29, 32] trials were assessed as unclear risk and 4 trials [20, 30, 31, 33] were assessed as low risk in the blinding of the outcome assessment. Of all these 11 trials, 7 trails [20, 21, 28, 29, 31, 32] were assessed as low risk and 4 [22, 27, 30, 33] were assessed as high risk, which was assessed in incomplete outcome data (Figure 2).

### 3.5. Publication Bias

**Figure 3:**
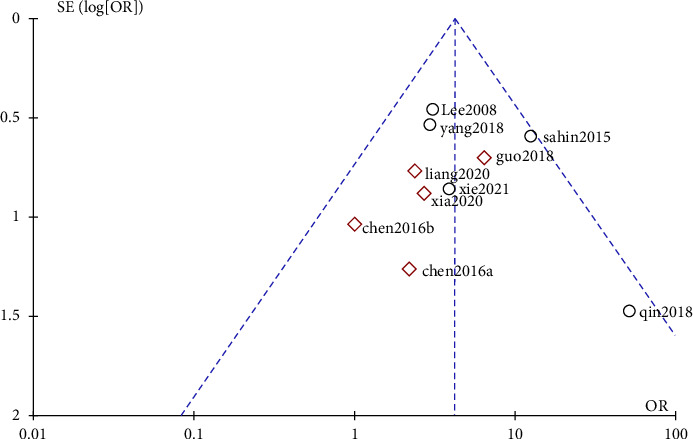
Publication bias of included studies.

As can be seen in Figure 3, all studies were not outside the funnel plot, but publication bias could be suspected based on the funnel plot asymmetry.

### 3.6. Trial Sequential Analysis

**Figure 4:**
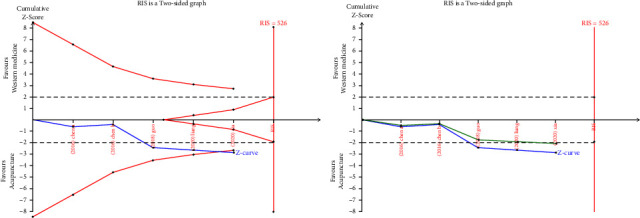
TSA on comparison of acupuncture verse western medicine in efficacy rate. The straight black line represents the conventional statistical boundary of *P*=0.05. The blue line indicates the cumulative *z*-score of the meta-analysis. The red line indicates the TSA boundary. The green line represents the *Z*-curve after the penalty statistic. RIS represents the required size of information.

Eleven RCTS [20–22, 27–33] reported the total clinical effective rate, which were analyzed sequentially, with a type I error of 5% and a statistical power of 80%. The information axis was set as the cumulative sample size, and the sample size was used as the expected information value (RIS).  Figure 4 [20, 21, 28, 29]shows that the *Z*-curve crosses the conventional boundary value and the TSA boundary value, indicating that the results obtained from this meta-analysis are robust and the efficacy of acupuncture in the treatment of CP and CPPS is positive. Meantime, the penalty curve also exceeded the traditional boundary value, which made the meta-analysis result more stable, but it did not reach the RIS value, and further research is needed.

**Figure 5:**
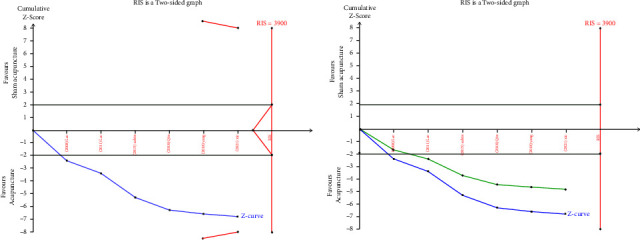
TSA on comparison of acupuncture verse sham acupuncture in efficacy rate. The straight black line represents the conventional statistical boundary of *P*=0.05. The blue line indicates the cumulative *z*-score of the meta-analysis. The red line indicates the TSA boundary. The green line represents the *Z*-curve after the penalty statistic. RIS represents the required size of information.

In Figure 5 [22, 27, 30–33], although the *Z* curve crossed the traditional boundary value curve and the penalty curve crossed the traditional boundary value curve, the *Z* curve did not cross the TSA boundary value curve and did not reach the RIS value, indicating that the results of meta-analysis may have false positive, and further research is needed to further prove the efficacy of acupuncture and sham acupuncture in the treatment of CP and CPPS.

### 3.7. Effect of the Interventions

Based on various outcome measures (pain score, urinary symptom, NIH-CPSI score, quality of life, and efficacy), different pooled data from 11 trials were used. The data were divided into stratified analyses according to different interventions of control groups. The effect of acupuncture on CP/CPPS is shown below, respectively.

#### 3.7.1. Acupuncture versus Western Medicine

**Figure 6:**
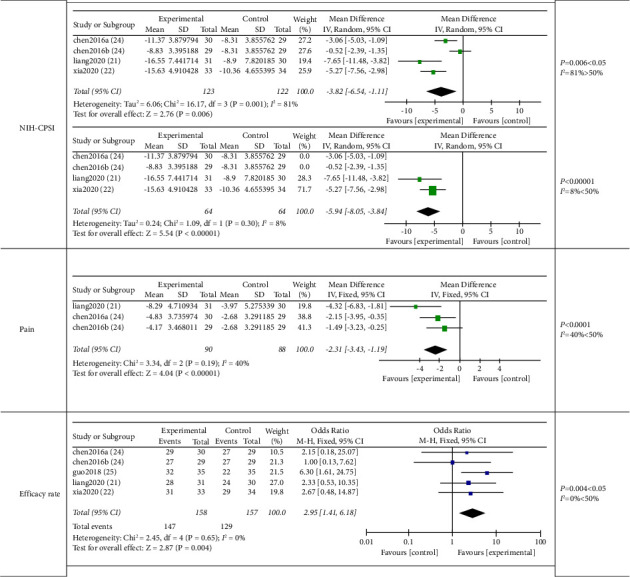
Meta-analysis on comparison of acupuncture verse western medicine in NIH-CPSI, pain, and efficacy rate.

A total of 4 studies [20, 21, 28, 29] including 1 three-arm trial [28] compared acupuncture with western medicine on CP/CPPS. It is obvious that acupuncture yielded the greatest benefits compared with western medicine in the NIH-CPSI score (−3.82 (95% CI, −6.54, −1.11), *P*=0.006, *I*^2^ = 81%), pain score [20, 28] (–2.31 (95% CI, −3.43, −1.19), *P* < 0.0001, *I*^2^ = 40%), and effective rate (2.95 (95% CI, 1.41, 6.18), *P*=0.004, *I*^2^ = 0%) [20, 21, 28, 29]. Only 1 study [20] reported the outcome of quality of life score (−1.98 (95% CI −3.12, −0.84), *P*=0.0007) and urinary symptom score, which had no significant differences between the two groups (−1.21 (95% CI, −2.48, 0.06), *P*=0.06). (Figure 6).

Sensitivity analysis: by analyzing the sources of NIH-CPSI heterogeneity, we found that the heterogeneity decreased after excluding 1 three-arm trial with the intervention of scalp acupuncture [28] (*I*^2^ = 8%, *P* < 0.00001).

#### 3.7.2. Acupuncture versus Sham Acupuncture

**Figure 7:**
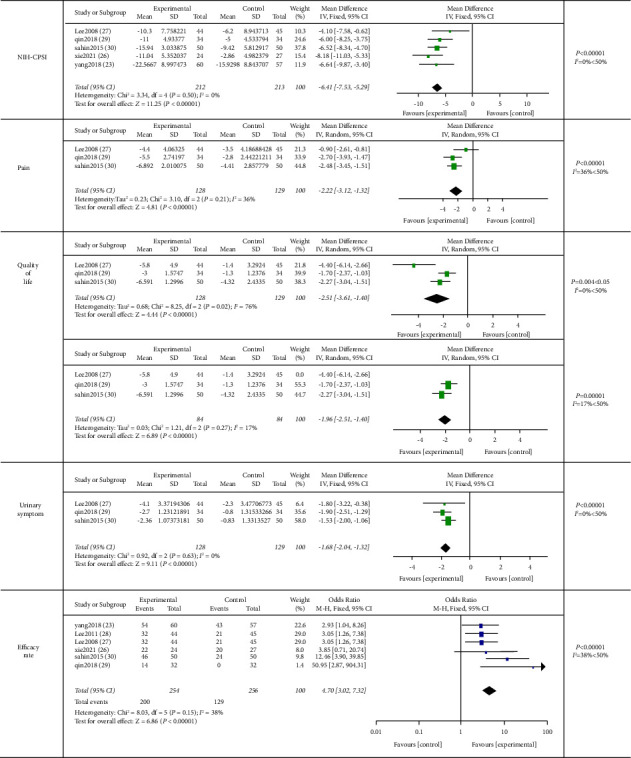
Meta-analysis on comparison of acupuncture verse sham acupuncture in pain, NIH-CPSI, quality of life, urinary symptom, and efficacy rate.

A total of 6 studies [22, 27, 30–33] compared acupuncture with sham acupuncture on CP/CPPS. Pooling of the data using a random effect model revealed that acupuncture could significantly improve the NIH-CPSI score (−6.41 (95% CI, −7.53, −5.29), *P* < 0.00001, *I*^2^ = 0%), pain score (−2.29 (95% CI, −2.99, −1.59), *P* < 0.00001, *I*^2^ = 36%), urinary symptom score (−1.68 (95% CI, −2.04, −1.32), *P* < 0.00001, *I*^2^ = 0%), the quality of life (−2.52 (95% CI, −3.64, −1.40), *P* < 0.0001, *I*^2^ = 76%), and effective rate (4.70 (95% CI, 3.02, 7.32), *P* < 0.00001, *I*^2^ = 38%) compared to sham acupuncture (Figure 7).

Sensitivity analysis: the source of the quality of life heterogeneity was analyzed. We found that the heterogeneity decreased (*I*^2^ = 17%, *P* < 0.00001) after eliminating 1 study with the frequency of 3 d/w [32].

## 4. Discussion

There were 10 studies, including 1 three-arm trial, with 798 patients included in the total. Acupuncture, as one of the traditional Chinese medicine therapies, includes many different acupuncture methods. In this meta-analysis, standard acupuncture and moxibustion, electroacupuncture, Tiaoshen acupuncture, scalp acupuncture, acupuncture combined with western medicine, and acupoint injection were all classified as broad acupuncture therapy (AT). All of them have been evaluated and compared with western medicine and sham acupuncture.

The overall quality of these identified trials was high (JADAD ≥ 4). The overall trials were at low risk. All studies have reported random sequence generation and prespecified expected outcomes without missing data. However, some studies were recorded deficiently about the outcome of blinding of participants and outcome assessment, and meantime, it was hard to judge the existence of the important other bias due to the original data were not available.

Despite the methodological quality and risk of bias limitations, acupuncture (AT) displayed a superior effect in the improvement of pains, urinary symptom, quality of life, NIH-CPSI score, and efficacy than western medicine (WM) and sham acupuncture (SAT). We found that in the acupuncture versus western medicine group, the heterogeneity in NIH-CPSI decreased after excluding 1 study (*I*^2^ = 8%, *P* < 0.00001) [28], of which the intervention was scalp acupuncture, while the others were manipulated by body acupuncture. Our findings indicate that different acupuncture positions might be the sources of the heterogeneity. Besides, we found that there are no significant differences in improving urinary symptom scores between the two groups (*P*=0.06), which was contrary to the original study. We speculated this inconsistency might be related to the process of raw data for being failed to contact the author. As for the results of quality of life in the acupuncture versus sham acupuncture group, the heterogeneity decreased (*I*^2^ = 17%, *P* < 0.00001) after eliminating 1 study [32]. According to Figures 2 and 7, the frequency of this study was 3 d/w, but other studies <3 d/w, which would be related to the high heterogeneity, and we speculated the frequency ≥3/w was more effective.

Meanwhile, to confirm the stability of the results of this meta-analysis, a trial sequential analysis was performed.  Figure 4 [20, 21, 28, 29] shows that the *Z*-curve crosses the conventional boundary value and TSA boundary value, although it does not reach the RIS value, indicating that the results obtained in this meta-analysis are robust. Compared with western medicine, the efficacy of acupuncture in the treatment of CP and CPPS is positive, and the penalty curve also exceeds the traditional boundary value, making the results of the meta-analysis more stable. In Figure 5, [22, 27, 30–33], although the *Z*-curve crosses the traditional boundary value curve and the penalty curve crosses the traditional boundary value curve, the *Z* curve does not cross the TSA boundary value curve, indicating that there may be false positive results in the meta-analysis, and further research is needed to further prove the efficacy of acupuncture and sham acupuncture in the treatment of CP and CPPS. However, unfortunately, the *Z*-curve did not exceed the RIS value in both analyses, indicating that a lot of research is needed in this regard.

Because the etiology and pathophysiological mechanism of chronic prostatitis are still unclear, current reports suggest that the pathogenesis of CP/CPPS may be related to abnormal immune response [34], central sensitivity [35–37], oxidative stress [38], pelvic floor muscle spasm [39] and neuropsychology [40]. Currently, there is no clinically clear treatment for CP/CPPS. The traditional main measures of CP/CPPS include taking anti-inflammatory drugs, antibiotics, and *α* receptor blockers, but long-term use will lead to adverse reactions such as nausea, dizziness, gastrointestinal discomfort, and hypotension [41], and some drugs even have no obvious therapeutic effect [42, 43]. Therefore, patients turn to alternative therapies such as acupuncture and cognitive behavioral therapy, especially acupuncture, which has been recognized for its good efficacy and high acceptance.

Related studies have shown that acupuncture can significantly reduce the pain of CP/CPPS patients, improve the NIH-CPSI symptoms, and improve the quality of life in patients [44]; the reasons include the following three mechanisms: (1) acupuncture can reduce the expression levels of interleukin (IL-6, IL-8), tumor necrosis factor (TNF) -*α*, prostaglandin E2, interferon (IFN) -*γ* and other inflammatory factors [45–52]. Increasing the expression levels of anti-inflammatory factors IL-2 and IL-10 and increasing the levels of CD3+, CD8+, and CD25+ in peripheral blood immune cells of CNP patients can regulate their immune function [53]. (2) Persistent chronic pain can reduce gray matter volume [36, 54] and activation [55] in brain regions such as the anterior cingulate gyrus in CP patients. The research on the central mechanism of acupuncture in the treatment of CP is still unclear, but there have been a large number of studies on the central analgesic effect of acupuncture [56]. For example, acupuncture can inhibit the formation of synaptic plasticity and affect the central sensitization state, and regulate the mitogen-activated protein activation (MAPK) mainly cell signaling pathway by inhibiting glial cell activation, and downregulate the expression of TRPV1, purine receptor (P2X3), and endocannabinoid receptor. The central analgesic effect was comprehensively exerted [57]. (3) The increase of oxidative stress products leads to the sensitization of nerve endings, which is an important factor in the occurrence of CP [58, 59]. Among them, the release of substance P and the inhibition of *β*-endorphin release make CP patients continue to have pain symptoms [60, 61]. Acupuncture can reduce the release of substance P and inhibit the increase of *β*-endorphin secreted by immune cells, so as to alleviate the clinical symptoms of CP patients [62].

There are 4 studies [21, 22, 24, 28] that described the adverse events. In acupuncture and sham acupuncture groups, specific skin symptoms including mild subcutaneous bleeding and pain were reported, while in the western medicine groups, gastrointestinal symptoms including nausea, abdominal pain, dizziness, and low blood pressure were recorded. Compared to drugs, acupuncture was safer. The security of acupuncture is ensured.

This review indicated that acupuncture is of significant benefit for CP/CPPS by setting strict inclusion/exclusion criteria and controlling the methodology quality. However, there were several limitations in this review. First, the methodological flaw is blinding absence, including participant blinding, therapist blinding, and assessor blinding, and researchers should pay more attention to the use of blinding in the future. Second, the data were extracted from 10 high-quality studies (JADAD ≥ 4) (Table 1), while most of them were with small samples (*n* < 50), and larger sample size high-quality studies are needed to confirm the effectiveness of acupuncture. Third, it can be seen from feature Table 2 that the acupoints and the number of acupuncture points in the 10 RCT are different; for example, Xia 2020 has 14 points, and Chen 2016 has 21 points, but Yang 2018, Xie 2021, Lee 2008, and Lee 2011 only have 4 points. Because of the small number of included literature and no stratification, the choice of acupoint may have an impact on the results of meta-analysis. In the future, high-quality literatures can be accumulated for further exploration. Fourthly, in this meta-analysis, we did not analyze the efficacy of acupuncture and different drugs (such as tamsulosin capsules, levofloxacin hydrochloride, or azithromycin capsules) on CP/CPPS, and new meta-analyses can be carried out on this point in the future. Fifthly, only 7 studies [20, 22, 27, 29, 31–33] reported the follow-up outcomes; thus, the longer-time efficacy of acupuncture for CP/CPPS needs to be explored further. Finally, this meta-analysis compared acupuncture with sham acupuncture or with western medicine. Unfortunately, the efficacy of different acupuncture treatments on CP/CPPS stays unclear, and a new meta-analysis in this regard can be conducted in the future.

## 5. Conclusion

This meta-analysis indicated that acupuncture (AT) has measurable benefits on CP/CPPS (pain, urinary symptom, quality of life, NIH-CPSI score, and efficacy rate), and the security of acupuncture was also ensured. However, methodological flaws, such as a small sample, the follow-up absence, and different kinds of acupuncture treatments exist, thus RCTs with larger sample sizes and longer-term observation are required to verify the effectiveness of acupuncture further, and a new meta-analysis can be conducted to analyze the efficacy of different acupuncture treatments on CP/CPPS in the future.

## Figures and Tables

**Table 1:** Characteristics summary of included studies.

				Treatment group				Control group			
Author/year (number)	Country language	Patients	Diagnostic criteria	Age	Sample size	Intervention	Frequency	Age	Sample size	Intervention	Frequency	Times	Outcomes	Positive or negative	Quality score (JADAD ≥ 4)
Liang 2020 [20]	China (China)	CP	① + ② + ④ + ⑥ + ⑦ + ⑧	42.2 ± 12.8	31	Electric acupuncture + western medicine	1/2d	41.9 ± 13.0	30	Western medicine	1/d	45	a+b + b1 + b2 + b3 + c + e	+	6
Xia et al. 2020 [21]	China (China)	CP/CPPS	① + ② + ⑥ + ⑦ + ⑧	43.24 ± 6.23	33	Tiaoshen acupuncture	1/d	43.70 ± 6.25	34	Western medicine	2/d	24	a + b + e	+	4
Yang et al. 2018 [27]	China (China)	CP/CPPS	① + ② + ③ + ⑦ + ⑧ + ⑨	18–55	60	Acupuncture	3/w	18–55	57	Sham acupuncture	3/w	24	a + b + c + d	+	4
Chen et al. 2016a [28]	China (China)	CP/CPPS	① + ② + ⑥ + ⑧	18–48	30	Scalp acupuncture + western medicine	1/d	19–45	29	Western medicine	1/d	24	a + b	+	4
Chen et al. 2016b [28]	China (China)	CP/CPPS	① + ② + ⑥ + ⑧	18–47	29	Scalp acupuncture	1/d	19–45	29	Western medicine	1/d	24	a + b	_	4
Guo and Wang 2018 [29]	China (China)	CP/CPPS	① + ② + ④ + ⑥	28–48	37	Acupuncture + acupoint injection	1/d	24–49	37	Western medicine	1/d	10	a + e	+	5
Xie et al. 2021 [22]	China (English)	CP	① + ② + ⑦ + ⑧ + ⑨	35.17 ± 8.76	24	Acupuncture	3/w	35.48 ± 7.92	27	Sham acupuncture	3/w	20	a + b	+	5
S. H. Lee and B. C. Lee 2008 [32]	Malaysia (English)	CP/CPPS	① + ② + ③ + ⑦ + ⑧	≥20	44	Acupuncture	2/w	≥20	45	Sham acupuncture	2/w	20	a + b + b1 + b2 + b3 + c + e	+	5
Lee et al. 2011 [30]	China (English)	CP/CPPS	① + ② + ③ + ⑦ + ⑧	40.9 ± 11.0	44	Acupuncture	2/w	42.8 ± 9.4	45	Sham acupuncture	2/w	20	a	+	5
Qin et al. 2018 [31]	China (English)	CP/CPPS	① + ⑦ + ⑧ + ⑨	18–50	34	Acupuncture	3/w	18–50	34	Shan acupuncture	3/w	24	a + e	+	7
Sahin et al. 2015 [33]	Turkey (English)	CP/CPPS	① + ⑧ + ⑨	20–50	50	Acupuncture	1/w	20–50	50	Sham acupuncture	1/w	6	a + b + b1 + b2 + b3 + e	+	5

§: three-arm trial. Diagnostic criteria: symptoms: ①prostate or pelvic region pain history: persistent or recurrent discomfort or pain in the prostate or pelvic area more than 3 months; ②lower urinary tract pathologies or genitourinary pain in the prostate or pelvic area more than 3 months; ③sexual dysfunction: impotence, premature ejaculation, spermatorrhea, sexual desire decreases; physical examination; ④prostate palpitation: abnormal size, uneven surface, local tenderness, or a lot of prostate fluid flow; auxiliary examination; ⑤ultrasonic examination: a slightly deformed section but with no expansion, uneven and inconsistent capsule often accompanied with prostatolith, less or not a smooth echo of a prostatic capsule and less even or uneven internal echo; ⑥laboratory test: EPS (white blood cell >10/HP objective and reduced of lecithin capsule); NIH-CPSI: ⑦NIH-CPSI total score ≥15 scores; age: ⑧age ≥18; ⑨age ≤50. Outcomes: a. treatment efficacy; b. NIH-CPSI score; b1. pain score; b2. urinary symptom score; b3. quality of life score; c. IPSS score; d. recurrence rate; e. side-effect.

**Table 2:** The methods of acupuncture and chosen acupoints of the enrolled studies.

Author/year (number)	Acupuncture and acupoints
Liang 2020 [20]	Electro-acupuncture, 5 points, CV4 (Guanyuan), SP6 (Saninjiao, bilateral), and SP9 (Yinlinquan, bilateral)
Xia et al. 2020 [21]	Tiaoshen acupuncture, 14 points, HT7 (Shenmen), GV24 (Shenting), GB13 (Benshen, bilateral), BL24 (Qihai), CV4 (Guanyuan), CV3 (Zhongji), ST28 (Shuidao, bilateral), SP6 (Sanyinjiao), ST36 (Zusanli), BL63 (Jinmen), KI5 (Shuiquan), and LR3 (Taichong)
Yang et al. 2018 [27]	Acupuncture, 4 points, BL23 (Shenshu), BL33 (Zhongliao), BL35 (Huiyang), and SP6 (Sanyinjiao)
Chen et al. 2016 [28]	Acupuncture, 21 points, DU24 (Shenting), DU22 (Xinhui), DU21 (Qianding), DU20 (Baihui), BL6 (Chengguang), BL7 (Tongtian), BL8 (Luoque), DU18 (Qiangjian), DU17 (Naohu), BL9 (Yuzhen), BL10 (Tianzhu), CV3 (Zhongji), CV4 (Guanyuan), ST29 (Guilai), KI12 (Dahe), ST31 (Biguan), ashi points, BL28 (Pangguangshu), BL32 (Ciliao), BL54 (Zhibian), and GB30 (Huantiao)
Guo and Wang 2018 [29]	Acupuncture, 7 points, CV4 (Guanyuan), SP6 (Sanyinjiao), CV3 (Zhongji), SP10 (Xuehai), ST36 (Zusanli), BL20 (Pishu), BL23 (Shenshu), acupoint injection, 2 points, BL54 (Zhibian), and BL32 (Ciliao)
Xie et al. 2021 [22]	Acupuncture, 4 points, BL23 (Shenshu), BL33 (Zhongliao), BL35 (Huiyang), and SP6 (Sanyinjiao)
S. H. Lee and B. C. Lee 2008 [32]	Acupuncture, 4 points, CV4 (Guanyuan), CV1 (Huiyin), SP6 (Sanyinjia), and SP9 (Yinlingquan)
Lee et al. 2011 [30]	Acupuncture, 4 points, CV4 (Guan Yuan), CV1 (Huiyin), SP6 (Sanyinjiao), and SP9 (Yinlingquan)
Qin et al. 2018 [31]	Acupuncture, 5 points, BL33 (Zhongliao, bilateral), BL23 (Shenshu), BL35 (Huiyang), and SP6 (Sanyinjiao)
Sahin et al. 2015 [33]	Acupuncture, 7 points, BL33 (Zhongliao), BL34 (Xialiao), BL54 (Zhibian), CV1 (Huiyin), CV4 (Guanyuan), SP6 (Sanyinjiao), and SP9 (Yinlingquan)

## Data Availability

All relevant data are within the manuscript and its supporting information file.

## Abbreviations

CP: Chronic prostatitis
CPPS: Chronic pelvic pain syndrome chronic prostatitis
RCT: Randomized controlled trial
AT: Acupuncture
WM: Western medicine
SAT: Sham acupuncture
NIH-CPSI: The National Institutes of Health Chronic Prostatitis Symptom Index.
